# Ribozyme Mediated gRNA Generation for *In Vitro* and *In Vivo* CRISPR/Cas9 Mutagenesis

**DOI:** 10.1371/journal.pone.0166020

**Published:** 2016-11-10

**Authors:** Raymond Teck Ho Lee, Ashley Shu Mei Ng, Philip W. Ingham

**Affiliations:** 1 Developmental and Biomedical Genetics Laboratory, Institute of Molecular and Cell biology, Agency of Science, Technology and Research (A-STAR), Singapore; 2 Lee Kong Chian School of Medicine, Nanyang Technological University, Singapore; Osaka University, JAPAN

## Abstract

CRISPR/Cas9 is now regularly used for targeted mutagenesis in a wide variety of systems. Here we report the use of ribozymes for the generation of gRNAs both in vitro and in zebrafish embryos. We show that incorporation of ribozymes increases the types of promoters and number of target sites available for mutagenesis without compromising mutagenesis efficiency. We have tested this by comparing the efficiency of mutagenesis of gRNA constructs with and without ribozymes and also generated a transgenic zebrafish expressing gRNA using a heat shock promoter (RNA polymerase II-dependent promoter) that was able to induce mutagenesis of its target. Our method provides a streamlined approach to test gRNA efficiency as well as increasing the versatility of conditional gene knock out in zebrafish.

## Introduction

Clustered Regularly Interspaced Short Palindromic Repeats (CRISPR)/Cas9 technology has revolutionized biology by greatly simplifying targeted mutagenesis [1]. The method relies on selection of the target sequence by the Cas9 DNA endonuclease, using a guide RNA (gRNA). The gRNA usually contains 20 nucleotides that are complementary to the target site as well as sequences that are recognized by Cas9 itself. Cleavage of DNA is mediated by Cas9 and requires the presence of a protospacer adjacent motif (PAM) that is downstream of the target sequence (N20NGG). Mutagenesis of the target site is mediated by aberrant DNA repair after cleavage.

Zebrafish researchers have been quick to adopt CRISPR/Cas9 technology for the generation of germ line mutations in their model system [1,2]. Typically mutations are induced by injection of both Cas9 mRNA and the relevant gRNA into newly fertilized egg. Target selection is limited by the nucleotide sequence of the transcriptional start site in bacteriophage promoters used to generate the gRNAs. Tolerance of mismatches in the first two nucleotides of the target site by Cas9 has been exploited to increase the number of target sites; however, some studies have shown that this decreases the mutagenesis efficiency [3,4]. Although Csy4, a sequence specific RNA endonuclease has been used successfully to increase the number of available target sites by incorporation of its recognition sites within the RNA transcript, however its toxicity precludes its application to zebrafish [4].

A potentially powerful extension of the application of CRISPR/Cas9 technology is the generation of somatic mutations by the *in-vivo* expression of gRNAs. Current methods use the zebrafish RNA polymerase (RNAP) III-dependent U6 promoter, which produces transcripts with well-defined start and end sites, to express gRNA in zebrafish [5,6]. The utility of this approach is limited by the fact it is believed that U6 transcripts start with a G, constraining the available target sites [5]. However a study using mouse U6 promoter have shown that transcripts start with a purine (A or G) [7]. Moreover, the U6 promoter is expressed ubiquitously; so temporal and spatial specificity of *in-vivo* mutagenesis can only be achieved by expressing the Cas9 endonuclease under the control of tissue-specific RNAP II promoters. The use of similar promoters to drive gRNA expression would greatly increase the versatility of CRISPR/Cas9 for conditional gene knock out; however, unlike the bacteriophage and RNAP III promoters currently used to generate gRNAs in vitro and in vivo respectively [5,8] the extensive processing of transcripts produced from RNAP II dependent promoters has precluded their use for gRNA expression [9–11].

Ribozymes are RNA molecules with catalytic activity and catalysis occurs utilizing sequence specific interactions within the RNA molecule [12]. In particular, Hammerhead (HH) and hepatitis delta virus (HDV) ribozymes are small and mediate sequence-specific intramolecular RNA cleavage [13]. Hammerhead and HDV ribozymes mediate cleavage of the RNA at the 3’ and 5’ end respectively. Incorporation of these ribozymes into RNA allows the generation of transcripts with precisely defined ends, an approach that has been successfully used for the *in-vivo* expression of gRNAs using RNAP-II dependent promoters both in yeast and mammalian cell lines [10,14]. In this study, we describe the application of ribozymes to generate gRNA both *in-vitro* and *in-vivo* for genome modification in zebrafish. Our method streamlines the generation of generation of gRNAs and increases the versatility of CRISPR/Cas9 mediated conditional mutagenesis in the zebrafish.

## Materials and Methods

### Fish husbandry and microinjection

Fish were maintained in the IMCB zebrafish facility and embryos were obtained through natural crosses and staged according to Kimmel et al. [15]. All experiments in this study were approved by the Institutional Animal Care and Use Committee (IACUC) of the Biological Resource Centre (BRC) (IACUC # 110638/151020). This was in accordance with guidelines set by National Advisory Committee for Laboratory Animal Research (NACLAR) and Agri-Food and Veterinary Authority of Singapore (AVA). All experiments were limited to 0–5 dpf zebrafish embryos. The *Tg(smyhc1*:*GFP)*^*i104*^ transgenic line has been previously described [16].

For CRISPR mutagenesis, embryos were injected with 200 fg Cas9 mRNA and 50 fg gRNA or 300 fg mRNA containing mCherry and gRNA (this reflects the amount of RNA injected in each embryo). To test the efficiency of DNA constructs expressing gRNA, 30 fg of plasmid and 20 fg Tol2 RNA was injected into embryos. *Tg(smhyc1*:*GFP)* ^*i104*^ was made using PI-*Sce*I therefore Tol2 transposase should not remobilize the transgene. Transgenic zebrafish were generated by injecting embryos with 30 fg Tol2 and 30 fg Plasmid DNA.

### Immunostaining and microscopy

Whole mount immunohistochemistry was performed as previously described [17]. Primary antibodies were: chicken anti-GFP (1:500; ab13970, Abcam), rabbit anti-DsRed/mCherry (1:500; 632496, BD Biosciences), and mouse anti-engrailed (1:50, 4D9, DSHB). Donkey secondary antibodies used were Dylight 488 conjugated donkey anti-chicken (Jackson ImmunoResearch), Alexa 488 donkey anti-mouse, and Alexa 546 donkey anti-rabbit (Invitrogen). Images were acquired on a Zeiss LSM700 or LSM800 confocal.

### Construction of transgenic and RNA synthesis

PCR amplification for cloning was done using PrimeSTAR Max (Takara, Clontech). gBlocks containing the HH-gRNA and HH-gRNA-HDV constructs were synthesized by IDT (Integrated DNA technologies) and final constructs were assembled using Gibson assembly (E2611, NEB). Middle entry vector containing Cas9 was cloned from pCS2-nCas9n [18]. Larger constructs containing Cas9 and Ubiquitin promoter [19] were constructed by Gateway cloning using components from the Tol2 kit [20]. HSP: *smo* gRNA was made using the *hsp70l* promoter and the Tol2 destination vector containing *cmlc2*:*GFP* marker from the Tol2 kit. Plasmids will be made available in Addgene.

mRNA was transcribed using Sp6 mMessage mMachine kit (Ambion, Invitrogen). gRNA was synthesized using the MEGAshortscript T7 kit (Ambion, Invitrogen). RNA was purified by lithium chloride and ammonium acetate for the mMessage mMachine kit and MEGAshortscript kit respectively. Further purification of RNA containing ribozyme is not necessary. Ribozyme cleavage of RNA was determined by denaturing 10% PAGE. Briefly, samples were resuspended in 50% formamide, 10mM EDTA and heated to 70°C for 5 min and chilled on ice. Denaturing PAGE gels contain 1xTBE and 7 M urea. PAGE Gel was pre-run at 20 W for 15 min before loading samples. Electrophoresis was carried out at 10 W.

### Genotyping and PAGE

PCR for genotyping was performed using GoTaq (Promega). Quantification of mutagenesis efficiency of various gRNA was performed as described [21]. Briefly, heteroduplex/homoduplex were induced by heating PCR products to 95°C for 5 min, 85°C for 10 s (ramp -2°C/s), 25°C for 10 s (ramp -0.1°C/s) and finally stored at 4°C. PAGE analysis was performed using 7.2% polyacrylamide gels in 1x TBE. Primers used for genotyping are listed in S1 Table. *smo* 5’ UTR F and *smo* ATG R were used to detect truncations in *smo* 5’ UTR.

For quantification of mutagenesis efficiency by cloning and sequencing, genomic DNA was extracted from twenty injected embryos and the gRNA target site was amplified by PCR (Smo 5’ UTR F and Smo 5’ UTR R) and cloned into pGEM-Teasy (Promega). Approximately twenty colonies were picked for colony PCR and amplified DNA was used for subsequent sequencing using Smo 5’ UTR R.

### Heat shock of *HSP*:*smo* gRNA

After injection of 250 fg Cas9 mRNA, embryos were shifted to 34.5°C when they reached 64 cell stage. Embryos were heat-shocked twice beginning at High stage for 2 h at 37°C with a 2 h interval at 34.5°C. After heat shock embryos were incubated overnight at 34.5°C.

## Results

### *In-vitro* generation of gRNA using Cis-acting hammerhead ribozyme

**Fig 1:**
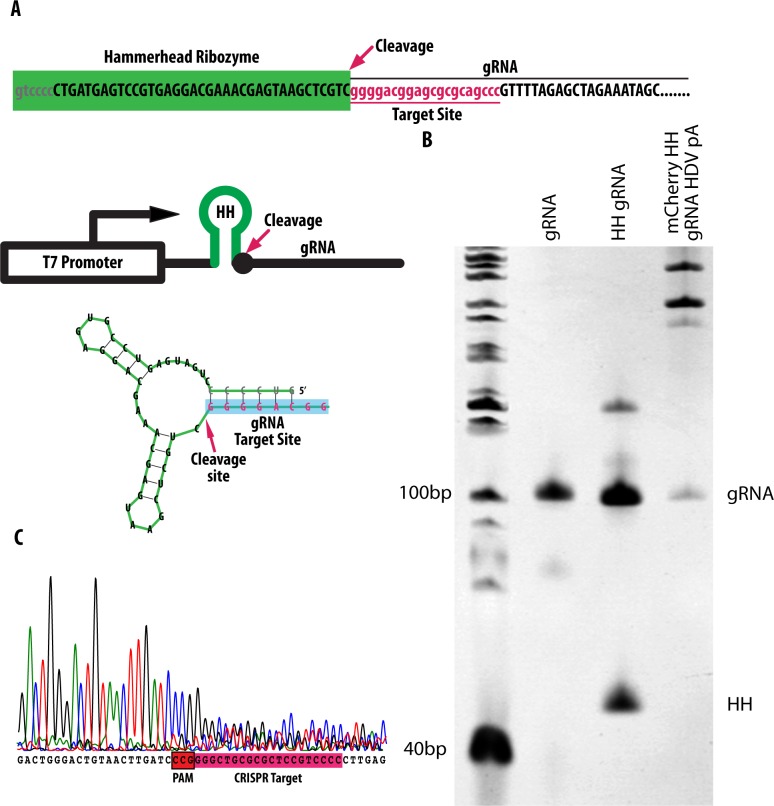
Structure and use of ribozyme to generate gRNA. (A) Sequence of HH ribozyme. Map of HH-gRNA. First 6 bases (grey text) of the ribozyme are complementary to the first six bases of the gRNA (red text) and cleavage occurs at the 5’ end of the gRNA (arrow). (B) Denaturing PAGE gel of IVT RNA. RNA containing HH and HDV ribozyme show spontaneous cleavage for generation of gRNA. HH and gRNA correspond to the HH ribozyme and gRNA respectively. (C) Sequencing of Smo 5’ UTR shows that the gRNA target site is mutagenized.

Hammerhead (HH) ribozymes are comprised of a RNA sequence motif that catalyzes self-cleavage from the 3’ end of the RNA molecule and they have been used to remove 5’ end heterogeneity of RNA transcripts. Cleavage of the HH ribozyme occurs at stem loop one (Fig 1A). To make use of a HH ribozyme to generate gRNA, the first six nucleotides of the HH ribozyme is made complementary to the first six nucleotides of the target site of the gRNA [14]. We refer to this as HH-gRNA.

It was previously shown that *in-vitro* transcribed (IVT) gRNA with ribozymes was able to guide Cas9 cleavage of its target in an *in-vitro* cleavage assay [14]. To verify whether HH could mediate release of the gRNA without affecting the *in-vivo* mutagenesis efficiency, a construct encoding HH-gRNA targeting the 5’ UTR of mRNA encoded by the zebrafish *smoothened* (*smo*) gene was generated. IVT of *smo* HH-gRNA showed the presence of a band corresponding to gRNA after electrophoresis (Fig 1B). Sequencing of individual embryos injected with this HH-gRNA and Cas9 mRNA, revealed mutations at the target site (Fig 1C).

### *In-vitro* generation of biscistronic RNA containing a fluorescent protein and gRNA

**Fig 2:**
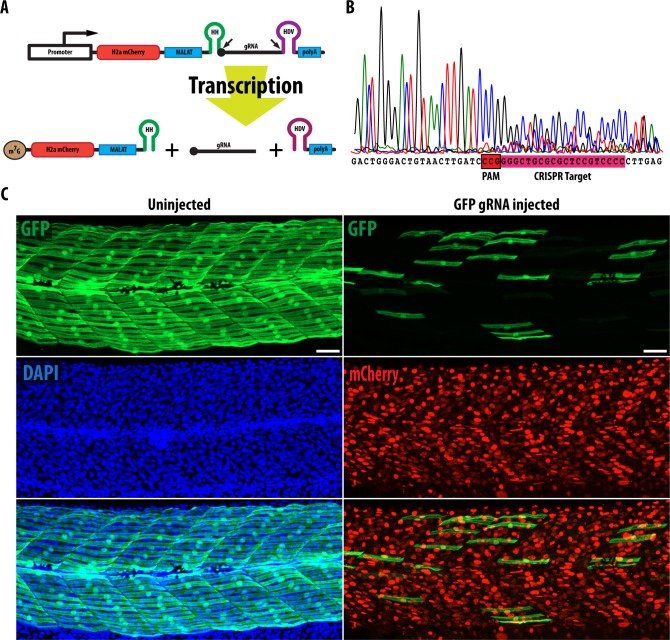
gRNA generated containing HH and HDV ribozyme is efficient at inducing mutagenesis of its target site. (A) mCherryHH-gRNA-HDV construct used to generate gRNA. H2a mCherry is used to label cells expressing gRNA and MALAT is used to stabilize the RNA after cleavage by HH and HDV ribozyme. (B) Injection of Smo gRNA together with Cas9 mRNA causes mutation of its target sequence. (C) Images of smyhc1: GFP embryos at 2dpf show that the majority of GFP expressing cells are lost upon injection of GFP gRNA and Cas9 mRNA. On average, there are ~24 GFP positive slow muscle fibers per somite in uninjected smyhc1: GFP embryos whereas only 6 GFP positive slow muscle fibres remained in each somite of injected smyhc1: GFP embryos (n = 5). Scale bars: 40 **μ**m.

To mimic transcript processing following transcription by RNAPol II, a new construct was generated with H2a mCherry and HDV ribozyme upstream and downstream of HH-gRNA (Fig 2A). This new gRNA-containing construct, referred to as mCherryHH-gRNA-HDV, was transcribed using the mMessage Kit (kit produces 5’ capped RNA for efficient translation). H2a mCherry was added as an independent assay for upstream sequence removal by the HH ribozyme as well as a visible marker of transgene-expressing cells in subsequent experiments. MALAT sequence forms a triple helical structure that stabilizes the H2a mCherry mRNA due to the removal of the polyA tail by ribozymes (Fig 2B and 2C) [10,22]. The HDV ribozyme does not require any base pairing with upstream sequences and mediates cleavage at its 5’ end. A previous study has shown that the sequence “UUUUU” can also terminate RNAP II transcripts [23] but this was not tested. IVT of the construct resulted in the detection of a band corresponding to gRNA following electrophoresis (Fig 1B). Zebrafish embryos injected with this transcript together with Cas9 mRNA showed nuclear accumulation of mCherry protein and harboured mutations of the target site in the *smo* gene (Fig 2B).

To validate general applicability of this method of ribozyme-mediated gRNA production, we tested another well-established gRNA that targets GFP [24]. Embryos carrying the *smyhc1*:GFP transgene that drives expression of GFP in slow-twitch muscle fibres were injected with mCherry-HH-gRNA-HDV targeting GFP along with Cas9 mRNA; all embryos (n = 34) showed a dramatic loss of GFP expression in their slow-twitch muscle fibres, reflecting efficient mutagenesis of the GFP target site (Fig 2C; S1B Fig, on average 6 GFP+ slow fibres remained in each somite compared to ~24 in wild type). This demonstrates the general utility of the HH ribozyme method for generating functional gRNA.

### Ribozyme mediated processing does not compromise gRNA efficiency

**Fig 3:**
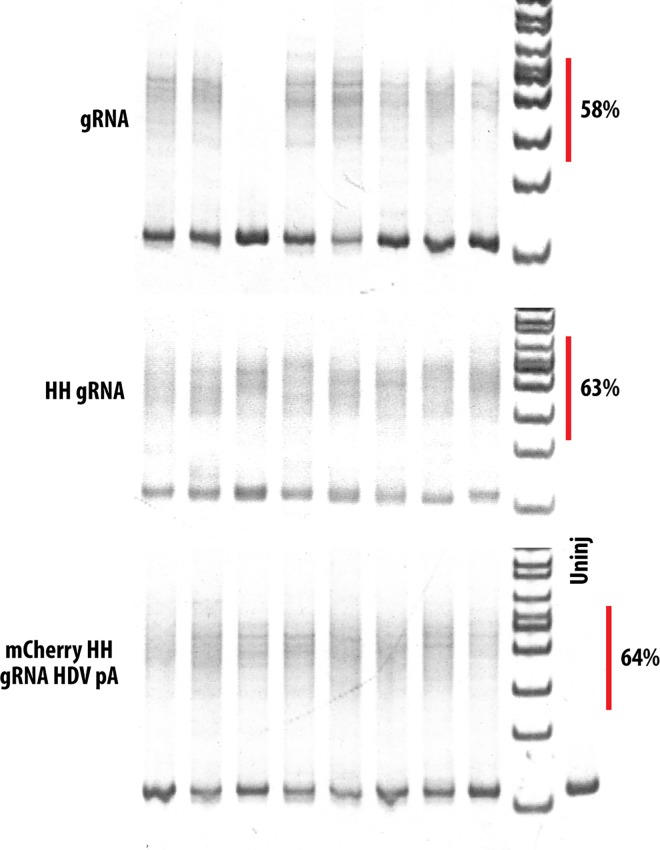
gRNA generated using ribozymes is as efficient as gRNA generated by transcription alone. PAGE analysis of smo 5’ UTR shows the formation of heteroduplex after injection of gRNA and Cas9 mRNA compared to the uninjected control. Comparison of intensity of heteroduplex and homoduplex shows that gRNA with or without ribozymes have similar efficacy in inducing mutagenesis. Red vertical bar shows the region of heteroduplex that was used for analysis. Percentage of heteroduplex vs homoduplex is shown.

To compare the efficiency of gRNA targeting the *smo* 5’UTR generated by ribozymes with that generated by the conventional method, we injected equimolar (equivalent to 20 fg of gRNA) of each gRNA together with 200 fg of Cas9 mRNA. Previously it was shown that polyacrylamide gel electrophoresis (PAGE) was a reliable method for the detection as well as quantification of subtle allelic alterations [21]. This method relies on the formation of heteroduplexes between different alleles, which migrate slower than homoduplexes during electrophoresis. Genomic DNA was extracted from single embryos and PCR was used to amplify the *smo* 5’UTR. To determine the efficiency of mutagenesis, genomic DNA was extracted from single embryos and smo 5’UTR was amplified by PCR. Efficiency of mutagenesis was determined by comparing the ratio of heteroduplex and homoduplex. We found that gRNA generated using ribozymes is as efficient as gRNA generated using conventional methods, both show ~60% efficiency (Fig 3). We also further evaluated this by cloning and sequencing gRNA target sites from pooled samples of twenty injected embryos. Sequencing of twenty clones from each sample showed that approximately 45% of these clones showed mutagenesis of its target site (S1A Fig). Therefore ribozyme mediated gRNA generation does not affect the efficiency of gRNA targeting.

### *In-vivo* expression of gRNA and Cas9 can induce target site mutation

**Fig 4:**
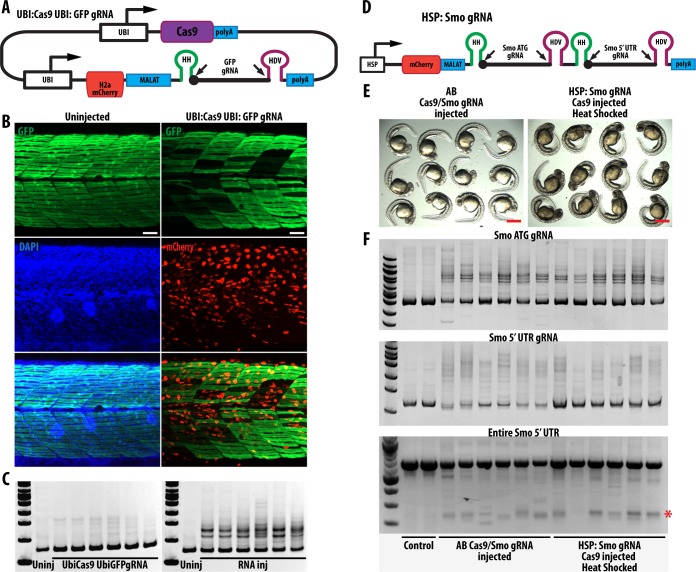
RNAPol II dependent promoters can be used to generate gRNA *in-vivo* and induce mutagenesis. (A) DNA Construct containing Cas9 and gRNA targeting GFP, both driven by ubiquitin promoter. (B) Transient expression of this construct in smyhc1:GFP embryos at 5dpf shows that some cells normally expressing GFP are lost. Cells expressing construct are labelled with H2a mCherry. On average 14 GFP positive slow muscle fibres remained in each somite after injections with this construct (n = 6), whereas there are ~24 GFP positive slow muscle fibers per somite in smyhc1: GFP embryos. Scale bars: 40 μm. (C) PAGE analysis of individual embryos injected with Ubi: Cas9 Ubi: GFPgRNA (left) and RNA of Cas9/GFP gRNA (right) show that GFP is mutagenized. (D) A heat shock promoter was used to drive the expression of two gRNA against Smo 5’ UTR. This construct was used to generate transgenic zebrafish (HSP: Smo gRNA). (E) Curved embryos seen after injection of AB with Cas9 mRNA and Smo gRNA targeting *smo* 5’ UTR and *smo* ATG (42 out of 117); and HSP: Smo gRNA embryos injected with Cas9 mRNA after heat shock (46 out of 131). Scale bars: 500 μm. (F) PAGE analysis of embryos from control (uninjected HSP: Smo gRNA) shows that Smo 5’ UTR and Smo ATG is mutagenized. Furthermore deletion of the region between the two gRNA targets can be detected (red asterisk).

As a preliminary test for the *in-vivo* expression of gRNA using ribozymes and RNAPol II dependent promoters, we generated a construct (UBI:Cas9 UBI:GFP gRNA) that drives ubiquitous expression of Cas9 and mCherryHH-gRNA-HDV targeting GFP (Fig 4A). This construct was injected into *Tg(smyhc1*:*GFP)*^*i104*^ embryos and expression of H2a mCherry was monitored to confirm expression of gRNA. We found a number of embryos (n = 24) with mosaic expression of H2a mCherry showing a restricted loss of GFP expression in slow-twitch muscle fibres, suggesting that expression of the construct could induce mutation of its target site (Fig 4B; S1B Fig, on average 14 GFP+ slow fibers remained in each somite compared to ~24 in wild type). However, loss of GFP expression was less extensive than that induced by RNA (14 compared with 6 in DNA and RNA injections respectively, Figs 2C and 4B; S1B Fig) implying that the *in-vivo* gRNA production from DNA expression constructs is less efficient. PAGE analysis confirmed that mutagenesis of GFP occurred (Fig 4C).

To explore further the expression of gRNAs by RNAPol II dependent promoters, another construct (HSP: Smo gRNA) expressing two gRNAs targeting Smo ATG and Smo 5’ UTR driven by the heat shock promoter (*hsp70l*) was generated (Fig 4D). As expected, heat shock of these embryos induced mCherry expression in the embryo (S2 Fig). Embryos carrying this transgene were injected with Cas9 mRNA and subjected to heat shock as described in Materials & Methods. At 30 hpf, a significant number (~30%) of these embryos exhibited curved body axes and U-shaped somites, phenotypes typical of *smo* loss of function mutants (Fig 4E; S3 Fig). Similar phenotypes were observed in embryos injected with Cas9 mRNA and *smo* gRNA targeting both *smo* 5’ UTR and ATG (42 out of 117, Fig 4E; S3 Fig). Heat shock of uninjected HSP: Smo gRNA embryos did not show any embryos with curved body (S3A Fig). Mutagenesis of gRNA target sites was confirmed using PAGE analysis and truncations of Smo 5’ UTR could also be detected (Fig 4F). Loss of *smo* activity leads to defects in muscle specification, including loss of Engrailed positive muscle pioneer cells and medial fast fibres [25,26]. Immunohistochemical analysis revealed effects on both cell types in the injected embryos (S3B Fig).

## Discussion

We have demonstrated efficient expression of gRNA using RNAP II dependent promoters without loss of targeted mutagenesis capacity. Conditional CRISPR/Cas9 mediated mutagenesis requires *in-vivo* expression of both Cas9 and gRNA; the ability to drive both using RNAP II dependent promoters should expand the versatility of the method significantly. For example, intersectional expression by different promoters for Cas9 and gRNA can be used to restrict gene targeting to very precise populations of cells as well as specific developmental stages. Moreover, the small size of ribozymes, allows expression of multiple gRNAs to increase the efficiency of mutagenesis or target multiple genes simultaneously.

Owing to the mosaic nature of DNA injections, far fewer heteroduplexes are seen following DNA injections compared to RNA injections (Fig 4C). This highlights the difficulty in validating gRNA targets using DNA injections. Low efficiency of mutagenesis might reflect low number of cells expressing constructs or failure of gRNA to mutagenize its target site. This also hampers comparisons between different gRNAs. By contrast, we find that RNA injections of gRNA and Cas9 are much more efficient. Combining ribozyme incorporation and IVT using bacteriophage promoters, allows testing of gRNA efficiency using RNA injections, before generating transgenic lines for conditional mutagenesis (S4 Fig).

In most cases where a single gRNA is used for mutagenesis of a gene of interest, mutations leading to frame-shifts or premature termination of the gene of interest are analysed for gene loss of function. There have been reports showing the low diversity of recovered mutant alleles, a number of which do not generate frame-shift mutations [3]. Therefore, it is possible that conditional alleles generated using CRISPR/Cas9 might lead to the formation of genetic mosaics in the tissue of interest. To circumvent this problem multiple gRNAs targeting the same gene might be employed to increase the likelihood of inactivation. Alternatively, generating conditional CRISPR/Cas9 transgenic in a heterozygous mutant background should increase the efficiency of inducing complete loss of function of the targeted gene.

Overall, our method provides a streamlined approach for generating conditional mutants, as the same gRNA can first be transcribed for testing mutagenesis efficiency and subsequently used for generation of stable zebrafish lines. Our method also provides a simple way to increase the repertoire of available gRNA targets.

## Supporting Information

S1 Fig Comparison of mutagenesis efficiency. (A) Sequencing results showing mutant alleles recovered from a pool of twenty injected embryos with the various gRNA constructs and Cas9 mRNA. gRNA target site is shown above with the PAM nucleotides shown in green. Red dashes and nucleotides in blue represent deletions and insertions respectively. Red line shows mutant clones after CRISPR/Cas9 mediated mutagenesis. (B) Table showing number of GFP+ slow twitch fibres remaining in WT, RNA of Cas9/GFP gRNA and Ubi: Cas9 Ubi: GFPgRNA injected embryos.
(TIF)

S2 Fig HSP: Smo gRNA express mCherry after heat shock. Embryos were heat shocked according to materials and methods section to test for expression of mCherry. Left panel show wild type embryos and right panel show HSP: Smo gRNA. Green asterisk show the expression of GFP in the heart from the cmlc: GFP used as the transgenesis marker. Embryos shown are 30 hpf. Scale bars: 500 **μ**m.
(TIF)

S3 Fig Characterization of Smo gRNA injected and HSP: Smo gRNA mutants. (A) Overview of control embryos (uninjected HSP: Smo gRNA), embryos injected with Cas9 mRNA and Smo gRNA, and heat shocked HSP: Smo gRNA embryos injected with Cas9 mRNA. Scale bars: 500 **μ**m. (B) Embryos were stained with Engrailed (Eng) to show defects in muscle specification when *smo* is mutagenesized. In the middle and right most panel, curved embryos were imaged and these embryos have defective formation of muscle pioneers and media fast fibers which are dependent on *smo* for its formation (Embryos shown are representative of the variation in observed phenotypes). Furthermore, these embryos have U-shaped somites (dotted line showing outline of somite in DAPI images) typical of lost of hedgehog signalling. Scale bars: 40 **μ**m.
(TIF)

S4 Fig Protocol for the mutagenesis of zebrafish using ribozymes. (A) Workflow for the mutagenesis of zebrafish. If conditional mutagenesis is not the aim of the experiment, last two steps (marked with *) can be omitted. (B-C) Plasmid map and sequence of p3E U6 HH gRNA plasmid. This vector can be used for gateway cloning. Primers used for designing gRNA and template for IVT are shown (C). As mentioned before HH ribozyme requires complementary sequence to the gRNA target sequence (shown by yellow box).
(TIF)

S1 File Supporting Information. (DOCX)

S1 Table Primers used for genotyping. (DOCX)
