# Supplementary figures and images for: Ribozyme Mediated gRNA Generation for In Vitro and In Vivo CRISPR/Cas9 Mutagenesis
Source: PLoS One. 2016 Nov 10;11(11):e0166020. doi: 10.1371/journal.pone.0166020 (PMC5104441; doi:10.1371/journal.pone.0166020)

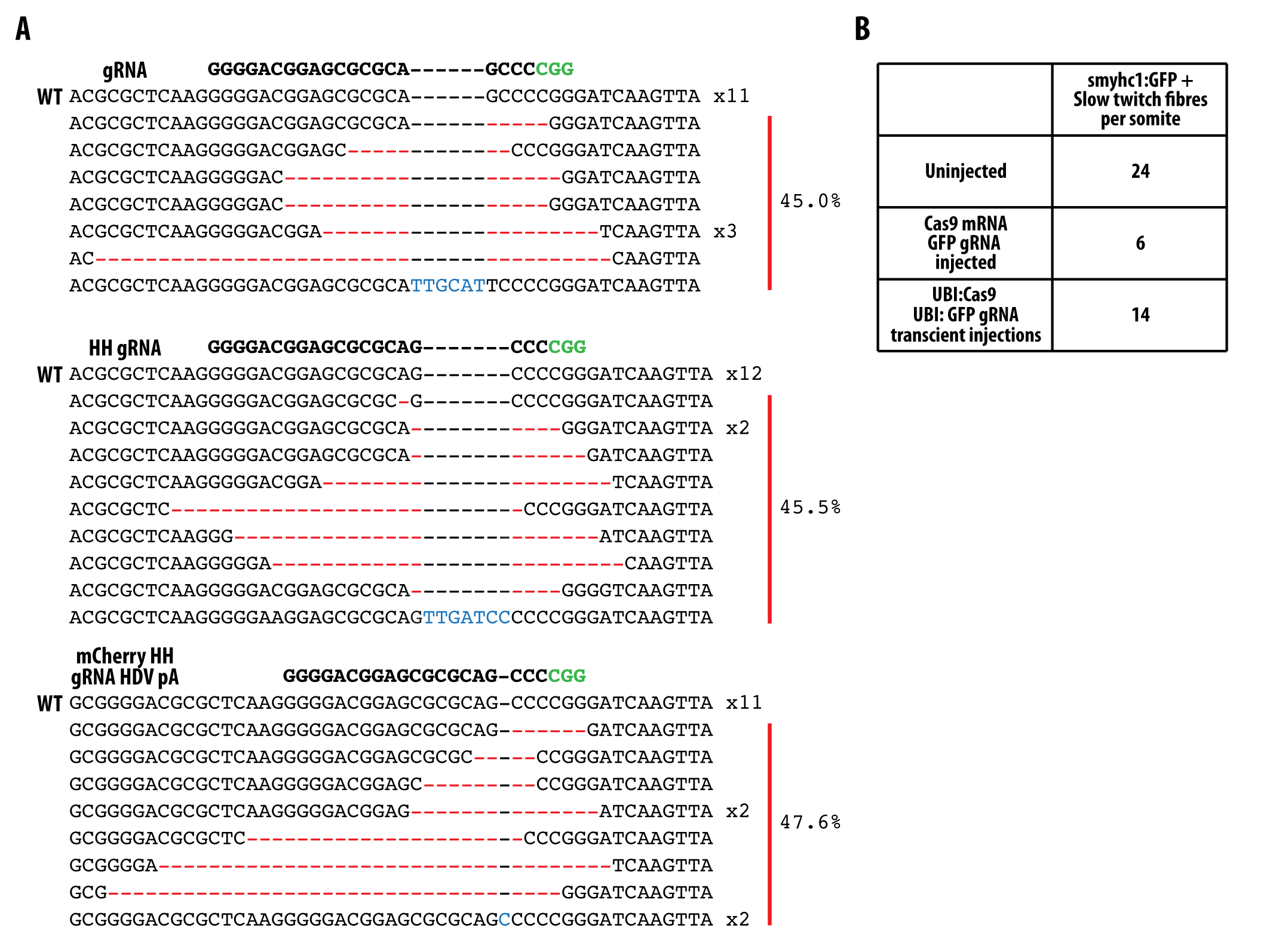

Supplement: S1 Fig — (A) Sequencing results showing mutant alleles recovered from a pool of twenty injected embryos with the various gRNA constructs and Cas9 mRNA. gRNA target site is shown above with the PAM nucleotides shown in green. Red dashes and nucleotides in blue represent deletions and insertions respectively. Red line shows mutant clones after CRISPR/Cas9 mediated mutagenesis. (B) Table showing number of GFP+ slow twitch fibres remaining in WT, RNA of Cas9/GFP gRNA and Ubi: Cas9 Ubi: GFPgRNA injected embryos. (TIF) [file pone.0166020.s001.tif]

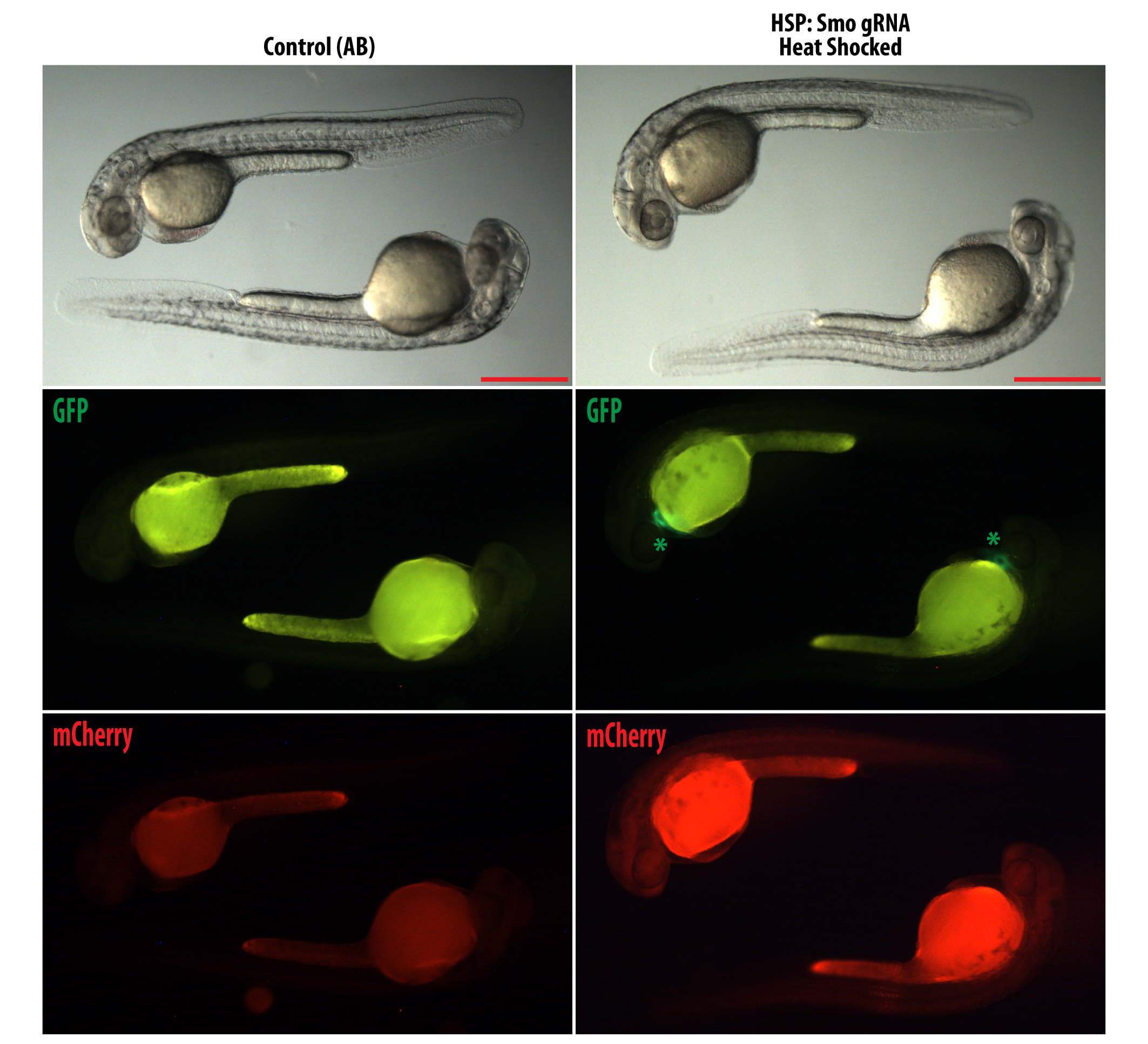

Supplement: S2 Fig — Embryos were heat shocked according to materials and methods section to test for expression of mCherry. Left panel show wild type embryos and right panel show HSP: Smo gRNA. Green asterisk show the expression of GFP in the heart from the cmlc: GFP used as the transgenesis marker. Embryos shown are 30 hpf. Scale bars: 500 μm. (TIF) [file pone.0166020.s002.tif]

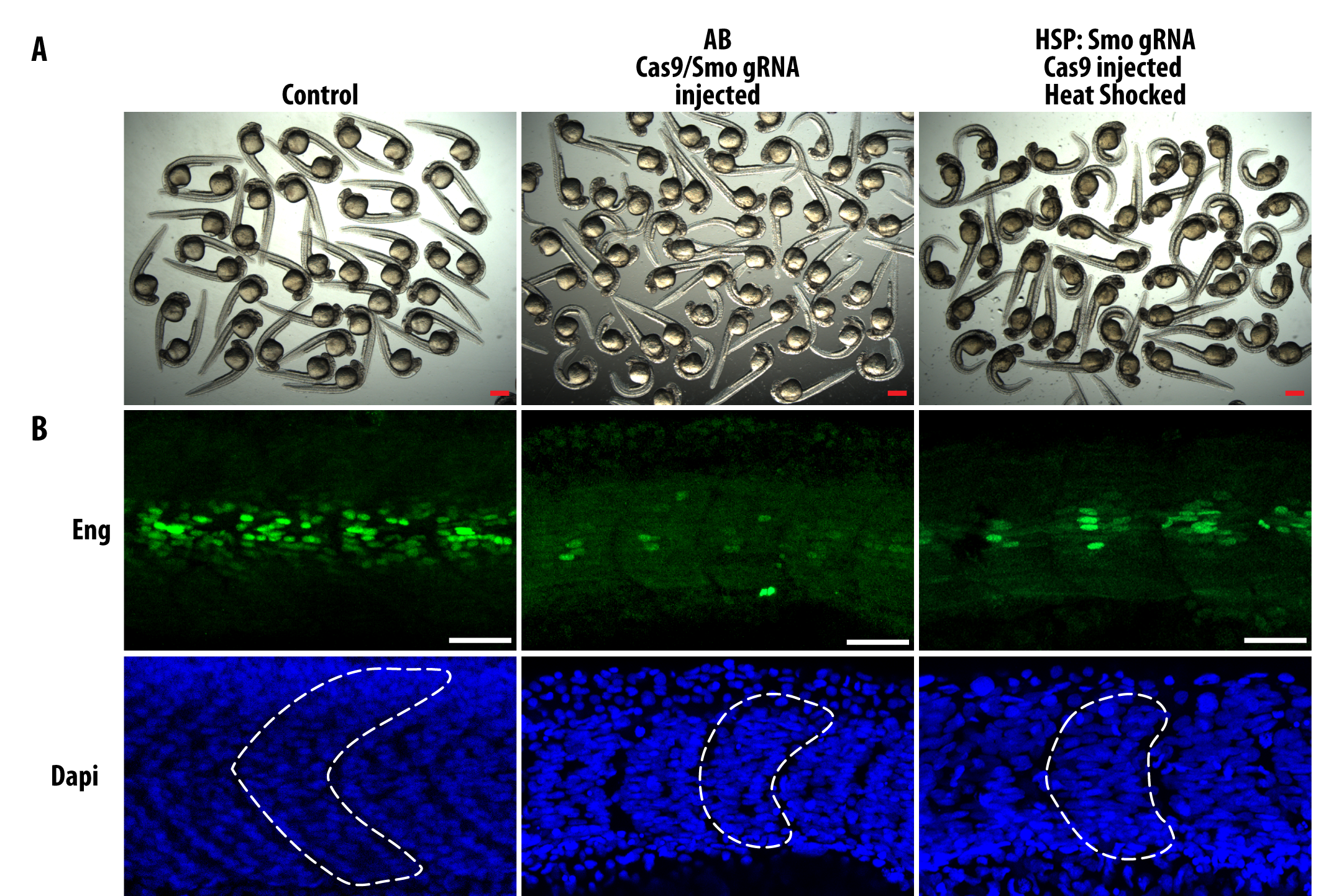

Supplement: S3 Fig — (A) Overview of control embryos (uninjected HSP: Smo gRNA), embryos injected with Cas9 mRNA and Smo gRNA, and heat shocked HSP: Smo gRNA embryos injected with Cas9 mRNA. Scale bars: 500 μm. (B) Embryos were stained with Engrailed (Eng) to show defects in muscle specification when smo is mutagenesized. In the middle and right most panel, curved embryos were imaged and these embryos have defective formation of muscle pioneers and media fast fibers which are dependent on smo for its formation (Embryos shown are representative of the variation in observed phenotypes). Furthermore, these embryos have U-shaped somites (dotted line showing outline of somite in DAPI images) typical of lost of hedgehog signalling. Scale bars: 40 μm. (TIF) [file pone.0166020.s003.tif]

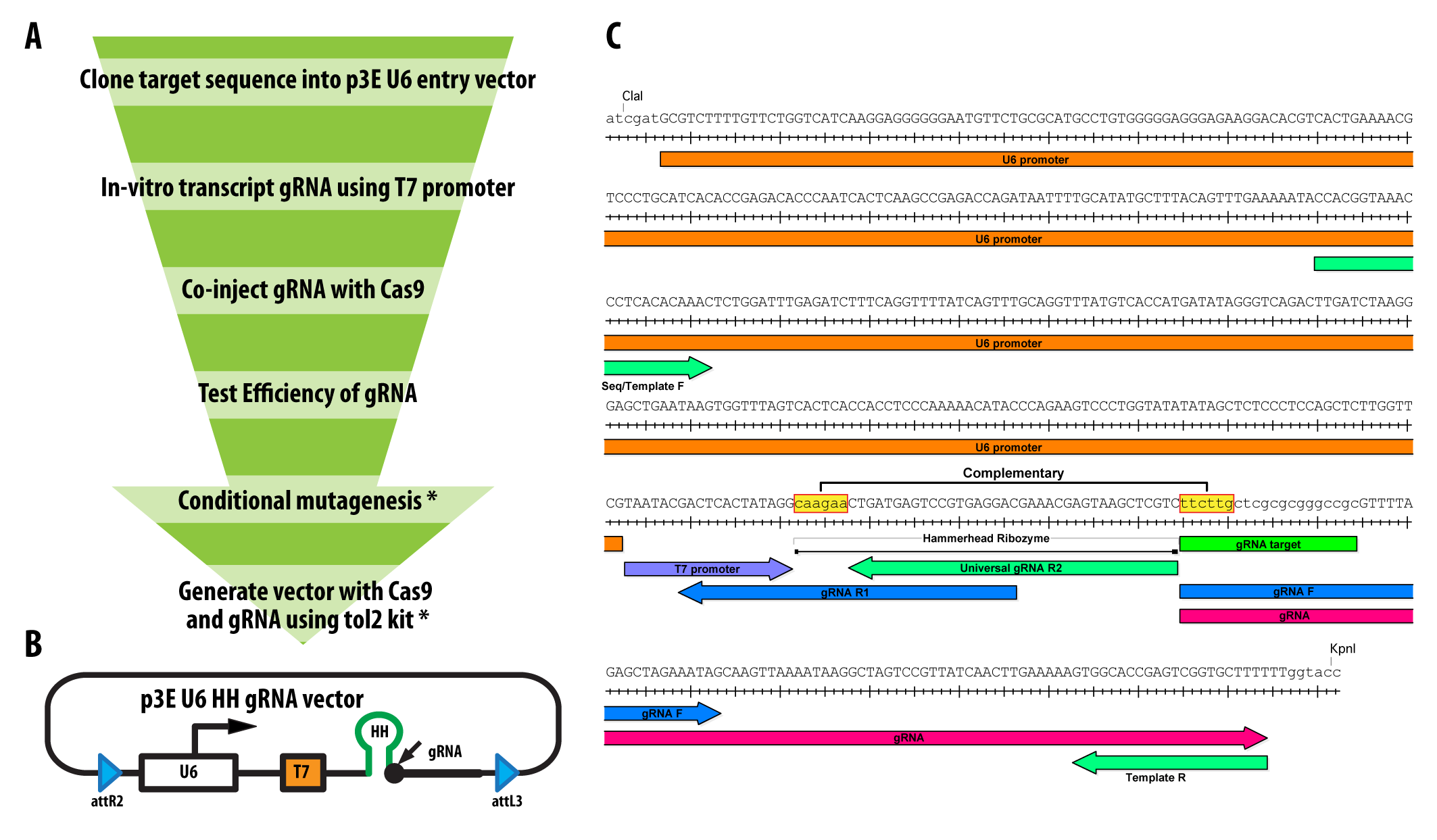

Supplement: S4 Fig — (A) Workflow for the mutagenesis of zebrafish. If conditional mutagenesis is not the aim of the experiment, last two steps (marked with *) can be omitted. (B-C) Plasmid map and sequence of p3E U6 HH gRNA plasmid. This vector can be used for gateway cloning. Primers used for designing gRNA and template for IVT are shown (C). As mentioned before HH ribozyme requires complementary sequence to the gRNA target sequence (shown by yellow box). (TIF) [file pone.0166020.s004.tif]
