# Supplementary material for: Ribozyme Mediated gRNA Generation for In Vitro and In Vivo CRISPR/Cas9 Mutagenesis
Source: PLoS One. 2016 Nov 10;11(11):e0166020. doi: 10.1371/journal.pone.0166020 (PMC5104441; doi:10.1371/journal.pone.0166020)
Supplement: S1 File — (DOCX) [file pone.0166020.s005.docx]

**Supporting information**

**Cloning of gRNA target vector**

To utilize ribozymes for generation of gRNA, two rounds of PCR followed by self-ligation is needed. First round of PCR utilize gRNA Primer F and gRNA Primer R1. PCR product is diluted and another round of PCR is performed with gRNA F and Universal gRNA R2. We typically use high fidelity PCR enzymes and 25 cycles of amplification. After second round of PCR, Dpn I is used to remove original template, before self-ligation. Refer to S1B and S1C Figs for map of template. After cloning, sequencing is used to verify that the cloning worked (primer used Seq/Template F). We typically use PCR with Seq/Template F and Template R to generate the template for IVT. We typically prefer to clone our gRNA constructs in vectors as it provides greater quality control and it is still quite fast (whole procedure typically only takes 2-3 days). Currently a p3E gateway plasmid containing the U6 promoter is available, this plasmid can also be used for IVT of gRNA. The U6 promoter can be replaced with other tissue specific promoters via cloning.

gRNA Primer F:

(N20)GTTTTAGAGCTAGAAATAG

N20 refers to the gRNA target sequence.

gRNA Primer R1:

GTCCTCACGGACTCATCAG(N6)CCTATAGTGAGTC

N6 refers to the reverse complement of the first nucleotides of the gRNA target sequence.

List of universal primers:

Universal gRNA R2 (5’ phosphorylated):

5’PO_4_/GACGAGCTTACTCGTTTCGTCCTCACGGACTCATCAG

Forward primer for sequencing and Template amplification:

CCACGGTAAACCCTCACACAAAC

Reverse Primer for template amplification:

AAAAGCACCGACTCGGTGCCAC

**Genotyping using PAGE analysis.**

We recommend PCR products that are between 100-500bp, typically we have used 6.9% PAGE gels. Running a wild-type or non-injected sibling control would be reveal whether there is any polymorphism, as they would also contain heteroduplex bands. Heteroduplexes can also be further analysed by sequencing. Heteroduplexes can be recovered by cutting out the bands and re-amplifying using the same primers. For detailed protocol refer to Chen et al. [21].
