# Supplementary material for: Ribozyme Mediated gRNA Generation for In Vitro and In Vivo CRISPR/Cas9 Mutagenesis
Source: PLoS One. 2016 Nov 10;11(11):e0166020. doi: 10.1371/journal.pone.0166020 (PMC5104441; doi:10.1371/journal.pone.0166020)
Supplement: S1 Table — (DOCX) [file pone.0166020.s006.docx]

| Smo 5’ UTR F | ACAACAATTCTCGCTTTCTGGTTTTACTG |
| --- | --- |
| Smo 5’ UTR R | GCCAAGATTTGCTCCGTTCCAG |
| Smo ATG F | GCACCGCAATTGTACGATGAACAG |
| Smo ATG R | AGGCGAGCCTAGGCACGTATTG |
| GFP Geno F | GAGCAAGGGCGAGGAGCTGTTC |
| GFP Geno R | GAAGTCGTGCTGCTTCATG |
